# Acute Myocardial Infarction (AMI) Treated with Snake Antivenom

**DOI:** 10.1155/2021/9945296

**Published:** 2021-10-16

**Authors:** Waleed Salem, Mohamed Gafar Abdelrahim, Layth Al Majmaie, Mohammed Dahdaha, Faten Al-Bakri, Amr Elmoheen

**Affiliations:** Emergency Department, Hamad General Hospital, Hamad Medical Corporation, Doha, Qatar

## Abstract

Cardiac complications following snakebites are uncommon but fatal. Here, we discuss a case of a snakebite that led to acute myocardial infarction (AMI). Forty-five-year-old male presented to the emergency room with snakebite on the right middle finger. He was given symptomatic treatment and admitted for observation. His vital signs and initial investigations were normal except for the white blood count that was high. During observation, he developed vomiting and bradycardia. He was diagnosed with a right bundle branch block on ECG. The patient developed chest pain after a few hours and was diagnosed with AMI on ECG. The toxicology team started antivenom therapy. His troponin kept rising initially but later started coming down without percutaneous intervention (PCI). He was treated successfully with antivenom therapy and discharged.

## 1. Introduction

Snakebite is a common presentation in many regions of the world and is associated with high morbidity and mortality [1]. The implications of snakebite are vast and are not limited to the affected area only. The venom is absorbed in the bloodstream and causes systemic symptoms as well [2]. Cardiotoxicity is one of the uncommon complications of snakebites, and people who present after a snake bite can develop myocardial infarction [3]. Many such cases have been reported [4]. Myocardial infarction can be multifactorial in such cases and can be a life-threatening complication of snake envenomation [3, 5]. The standard treatment is performing percutaneous intervention (PCI) after achieving hemodynamic stability and giving antivenom to the patient [3]. In this case report, we present a rare case in which the patient did not have to undergo PCI after he suffered from an acute myocardial infarction (AMI) following snakebite, and his condition improved after administering antivenom.

Elapid envenomation is rare in Qatar so that crotaline bites can be a concern. In the Middle East, including Qatar, the Saharan horned viper called Cerastes cerastes and Cerastes gasperettii are the most prevalent species of snakes [6].

## 2. Case Presentation

**Figure 1:**
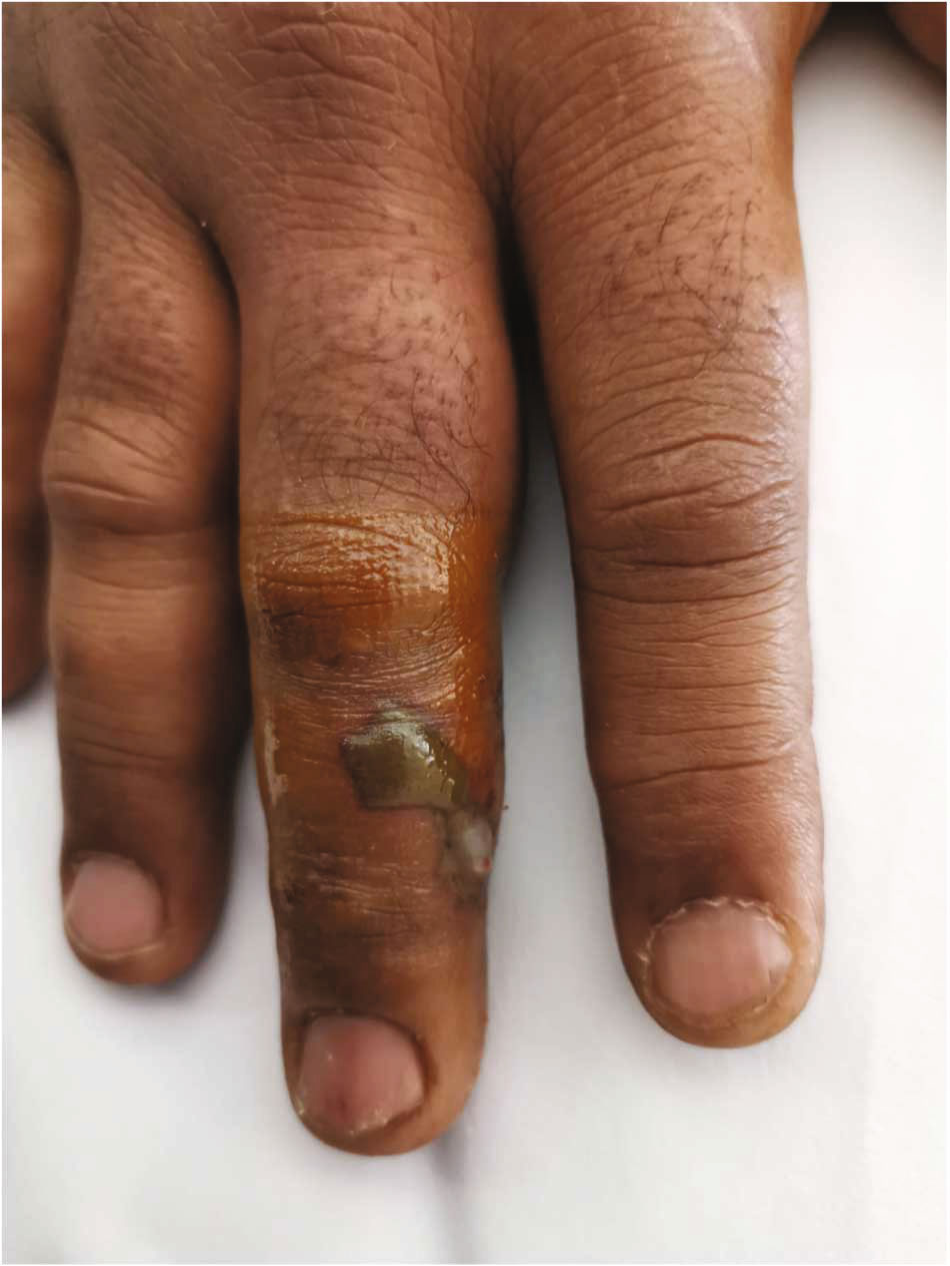
Snakebite of the middle finger.

**Figure 2:**
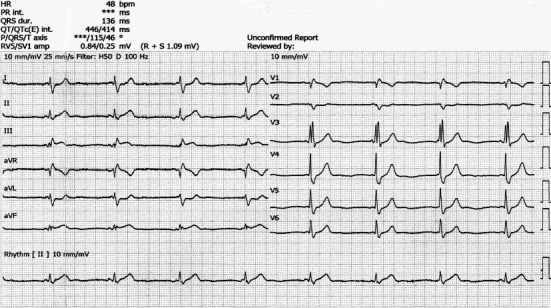
Electrocardiogram (ECG) shows right bundle branch block (RBBB).

**Figure 3:**
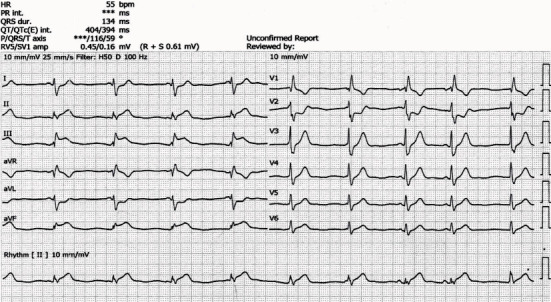
Electrocardiogram (ECG) shows ST segment elevation in the inferior leads.

A forty-five-year-old male patient presented to the emergency department on day 1 with a snakebite on his right middle finger followed by pain and swelling (Figure 1). He tied the area above his wrist after the bite. His vitals were normal on presentation, and blood investigations were sent. His white blood count was high, while the rest of the investigations were normal (Table 1). Then, the patient was admitted to the acute medical unit for observation, and IV fluid, hydrocortisone, and paracetamol were administered. On day 2 (after 16 hours from the envenomation), the patient started to have episodes of vomiting and started to be bradycardic with a heart rate of 50 beats per minute. His electrocardiogram (ECG) showed a right bundle branch block (RBBB) (Figure 2). Investigations were performed again, and they showed a reduction in platelet count and an increase in the international normalised ratio (INR). The patient's vitals were continuously being monitored; toxicology and medical ICU (MICU) teams were contacted. After 4 hours (20 hours from the envenomation), the patient had an episode of chest pain. The ECG was repeated and showed an ST segment elevation in inferior leads and ST segment depression in anterior chest leads (Figure 3). The patient had tachy-bradyarrhythmia; heart rate: 30-130 beats per minute, as well. The toxicologist advised to start antivenom (polyvalent) and monitor the patient for possible anaphylaxis.

Echocardiography showed normal-sized left ventricle, mild septal hypertrophy, and normal global systolic LV function (EF 56%) with no regional wall motion abnormality seen. The patient was given diphenhydramine 50 mg stat and fentanyl 50 mcg stat, and isosorbide dinitrate (ISDN) infusion 12.5 mcg was started. Blood investigations including complete blood count (CBC), comprehensive metabolic panel (CMP), disseminated intravascular coagulation (DIC) panel, myoglobin, troponin, and lactate dehydrogenase (LDH) levels were sent. Myoglobin, troponin, and creatine kinase (CK) were high (Table 2).

The patient started on oral aspirin, clopidogrel, and heparin bolus of 5000 units intravenously, followed by 12 unit/kg/hr infusion. As the patient's condition was deteriorating due to multiple ongoing pathologies, including DIC and high troponin followed by inferior wall myocardial infarction (MI), he was admitted to the medical intensive care unit (MICU) at night of day 2. The patient was having premature ventricular contractions (PVCs) and ectopic beats. The patient also had severe chest pain, and high sensitive troponin T went up to 2700 ng/L from 1080 ng/L. He was given IV morphine 2 mg stat, and isosorbide dinitrate (ISDN) infusion was increased to 30 mcg from 12.5 mcg.

The toxicologist advised continuing polyvalent antivenom. The patient received a total of 8 vials.

On day 3, the patient's condition stabilized. He was pain-free with normal ECG and normal rhythm. Also, INR and fibrinogen normalized. However, troponin was still very high (nearly 3400 ng/L).

On day 5, the patient had a normal ECG, INR, and fibrinogen. His troponin level also started coming down (Table 2). Anticoagulation was stopped at this point as platelet count was low. On day 6, the patient was started on low-dose bisoprolol and stepped down to the ward.

Computed tomography (CT) coronary angiogram was performed after two months as an out-patient, and the result was not significant for coronary artery disease. Because of the reassuring result, the cardiologist decided not to perform percutaneous coronary intervention (PCI).

## 3. Discussion

Myocardial infarction (MI) is a fairly uncommon complication of snake bite. Its pathophysiology is complex and multifactorial [3]. It has been reported in many cases of snakebite with no previous history of any cardiac disease or other comorbidities that increase the risk of cardiac diseases like diabetes and hypertension [7]. This complication has also been reported in people with a previous history of cardiac disease even after stenting [8]. Many mechanisms have been proposed for cardiac complications and myocardial infarction caused due to snake envenomation [8]. However, it is often due to a mix of various factors, such as hypovolemic shock, anaphylactic shock, hypercoagulability, hyperviscosity, coronary spasm, and direct cardiotoxicity of the snake venom [5]. There can be one or more predominant factors of these that can lead to this complication. A case was reported where a known case of coronary artery disease with stenting suffered from a snake bite that was followed by myocardial infarction due to thrombosis of the stent [8]. In another similar case, a 73-year-old patient developed ST segment elevation myocardial infarction (STEMI) after snake envenomation due to thrombosis. The complication was treated by carrying out revascularization and stenting [9]. A case of a 50-year-old woman was reported who was bitten by a viper. Initially, she did not have any systemic complications at the start, but after five days of snakebite, she presented with thrombotic microangiopathy that caused myocardial infarction (MI) during the recovery phase. She was managed using low molecular weight heparin (LMWH) and antiplatelet drugs but could not survive due to further complications [10].

Thrombosis seems a common cause of myocardial infarction (MI) after a snake bite as the number of reported cases caused by thrombosis is higher. However, it is not the only cause. Another case report described a 40-year-old man who had snake envenomation, and myocardial infarction (MI) was later confirmed on investigations. However, there was no risk factor for coronary artery disease, and coronary arteries were found to be normal on catheterization. There can be different mechanisms involved in this case. The cardiac event can be due to direct cardiotoxicity of the snake venom [11]. Similarly, in another case, a young patient developed acute myocardial infarction (AMI) following a snakebite. His coronary arteries were found to be normal on catheterization, and a segmental contraction abnormality was noticed in cardiac tissue. This contraction abnormality can be due to the direct cardiotoxicity of snake venom [12].

The above-mentioned literature clearly shows that myocardial infarction (MI) is one of the complications of snakebite, and several pathological pathways can lead to it. However, the pathophysiology starts from the snake venom entering the bloodstream and leading to systemic complications due to toxins [13]. Cardiac complications are not common in snake envenomation but still occur. T wave abnormalities are most commonly observed in such cases. ECG changes are short-lived, but if they are persistent, then the reason is direct cardiotoxicity [14]. All other causes of myocardial infarction (MI) are also due to other complications caused by snake venom. The toxins present in snake venom can lead to disseminated intravascular coagulation (DIC), leading to thrombus formation that can eventually cause myocardial infarction [15]. Bleeding and hypovolemic shock can also occur following envenomation [16], and it is another cause of cardiac complications.

It is evident that snake venom is the cause of cardiac complications of snakebite. Therefore, many complications can be prevented by prompt treatment [17]. Moreover, studies show that administering antivenom can significantly reduce the damaging effects of snake toxins. In a study conducted on mice, hemorrhagic activity totally ended, and local muscle damage was prominently reduced after administering antivenom [18]. Different types of antivenoms have been tested and are found to be effective in neutralizing several effects of snake venom [19]. Such cases have been reported where the patient was managed conservatively by giving antithrombotic treatment and antivenom along with supportive treatment. Percutaneous intervention (PCI) was not needed at all [20]. In our case report, we also described how there was an improvement in patient's condition after antivenom administration, and the patient did not need percutaneous intervention (PCI). It is most possibly due to damage control and reversal brought about by antivenom therapy. Various next-generation antivenoms are now available that are capable of neutralizing the effect of different snake toxins [21]. Timely antivenom administration can be associated with improved outcomes even after the development of severe complications like myocardial infarction (MI), as witnessed in our case [22].

It is important to acknowledge that there are some possible limitations of using antivenom as a treatment of myocardial infarction in this case. Firstly, the causal relationship between clinical improvement and antivenin administration cannot be clarified in the absence of a control group. Time alone, rather than antivenin, might be responsible for the observed improvement. Secondly, the patient did not receive a diagnostic percutaneous coronary intervention (PCI), although his later CT coronary angiogram was not significant for coronary artery disease. Thirdly, the coronary angiogram is still needed in such cases, as it is the standard of care to treat a STEMI, whether or not there was a snakebite [23, 24].

## Figures and Tables

**Table 1:** The results of investigations carried out on presentation.

	Value *w*/SI units	Normal range
White blood cells (WBC)	14.2 × 10^9^/L	4.0 − 10.0 × 10^9^/L
Hemoglobin (Hgb)	173 gm/L	130-170 gm/L
Platelet	199 × 10^9^/L	150 − 400 × 10^9^/L
Urea	6.2 mmol/L	2.8-8.1 mmol/L
Creatinine	95 *μ*mol/L	62-106 *μ*mol/L
Sodium	142 mmol/L	136-145 mmol/L
Potassium	4.1 mmol/L	3.5-5.1 mmol/L
Troponin-T HS	7 ng/L	3-15 ng/L
C-reactive protein (CRP)	0.8 mg/L	0.0-5.0 mg/L
Lactic acid	1.0 mmol/L	0.5-2.2 mmol/L
pH (venous)	7.34	7.35-7.45
pCO2 Ven	44.7 mmHg	35-45 mmHg
HCO3-Ven	24 mmol/L	22-26 meq/L

**Table 2:** The results of cardiac markers carried out after the patient developed chest pain.

	Date	Value *w*/SI units	Normal range
Troponin-T HS	Day 6 04:07:00	2,425 ng/L	3-15 ng/L
	Day 5 05:02:00	2,551 ng/L	
	Day 4 04:05:00	2,309 ng/L	
	Day 3 17:04:00	2,405 ng/L	
	Day 3 04:57:00	2,424 ng/L	
	Day 2 23:48:00	3,065 ng/L	
	Day 2 17:41:00	3,439 ng/L	
	Day 2 11:41:00	3,407 ng/L	
	Day 2 02:32:00	2,783 ng/L	
	Day 1 20:01:00	1,089 ng/L	
	Day 1 00:57:00	7 ng/L	
Myoglobin	Day 1 20:35:00	467 ng/mL	28-72 ng/mL
Creatine kinase (CK)	Day 1 20:35:00	698 U/L	39-308 U/L

## Data Availability

Data are available on request.
